# Application of stacked bidirectional LSTM neural networks in reservoir porosity prediction

**DOI:** 10.1038/s41598-025-23095-8

**Published:** 2025-11-10

**Authors:** Peng Zhang, Shunhao Hu, Ying Xiao, Pang Chen, Dazhen Sun

**Affiliations:** 1 Geophysical-COSL, Geophysical R&D Institute, China Oilfield Services Ltd., Tianjin, 300459 China; 2 School of Geoscience and Technology, Southwest Petroleum University, Chengdu, 610500 China

**Keywords:** Petrophysical parameter prediction, Rock physics modeling, S-BiLSTM, Deep learning, Energy science and technology, Engineering, Mathematics and computing, Solid Earth sciences

## Abstract

Porosity directly affects the fluid storage and transportation capacity of reservoirs, and is an important parameter for portraying the physical properties of oil and gas reservoirs. However, existing porosity prediction methods have obvious limitations. Traditional petrophysical modeling and cross-plot method struggle to characterize nonlinear relationships under complex geological conditions. Machine learning methods such as support vector machines (SVM) and BP neural networks rely on manual feature extraction, resulting in limited generalization ability. These methods show significant accuracy degradation in noisy data, with insufficient noise immunity. For this reason, this study uses petrophysical modeling to generate large-scale and diverse synthetic data sets of elastic and anisotropic parameters based on field logging data. And an enhanced stacked bi-directional long short-term memory (S-BiLSTM) model is proposed, which uses stacking of bi-directional LSTM layers to enhance feature extraction and prediction capabilities. Validation is carried out using seismic field-measured data from an oil field in southwest China, and the results show that the method has high accuracy and robustness in porosity distribution prediction. Specifically, the prediction accuracy of the S-BiLSTM model reaches 92.19%, which is 7.81% higher than that of the LSTM model and 14.06% higher than that of the RNN model. It also performs well under noisy data. At a signal-to-noise ratio of 10dB its accuracy is 82.81% surpassing RNN at 59.38% and LSTM at 68.75%. When the signal-to-noise ratio drops to 1dB its accuracy remains 76.12% significantly higher than RNN at 44.81% and LSTM at 53.5%. This study explores the potential of deep learning in the prediction of reservoir physical properties parameters, which has certain application value for oil and gas exploration and development.

## Introduction

Reservoir evaluation is the core link in oil and gas exploration and development, and its core objective is to provide basic data support for reservoir modeling, reserve calculation and development plan design through quantitative analysis of physical parameters^1^. Porosity, as a core physical parameter of reservoir, not only directly determines the storage capacity of the reservoir, but also has a complex coupling relationship with permeability, water saturation and other parameters^2^. Its measurement methods include direct measurement which involves physically measuring porosity on core samples in a laboratory with the highest accuracy but high cost^3^ and indirect estimation which involves estimating porosity from downhole logging data seismic data or other geophysical methods combined with petrophysical models^4^. Indirect estimation needs to integrate geological and logging information to build prediction models but strong nonlinearity under complex geological conditions limits the accuracy of traditional methods^5,6^. Porosity prediction on seismic data is more complex than on log data due to lower resolution higher noise interference and greater uncertainty from multiple solutions in seismic inversion. Seismic data inherently contains noise from acquisition and processing which distorts the reflection signals related to porosity. Its vertical and horizontal resolution is insufficient to capture fine-scale porosity variations especially in heterogeneous reservoirs. Moreover, the mapping relationship between seismic attributes and porosity is affected by multiple factors including lithology fluid properties and subsurface inhomogeneity leading to ambiguity in prediction. Recently geostatistical inversion combined with data-driven methods^7^ and using seismic attributes^8^ has improved the accuracy of indirect estimation^9^.

Machine learning handles reservoir parameter relationships through nonlinear fitting SVM^10^, BP neural networks^11^ and others^12^ are widely used in porosity prediction, but they rely on manual feature extraction and have local optimization issues^13^. Recent optimization algorithms namely PSO-optimized SVM^14^ enhanced SVM’s performance in reservoir parameter prediction via particle swarm optimization. Gradient boosting models^15^ improved carbonate reservoir permeability prediction through refined tuning. Machine learning applications in heterogeneous carbonate gascondensate reservoirs^16^ have also achieved reliable permeability prediction results. A parametric study^17^ validated machine learning’s effectiveness in reservoir permeability prediction. Tree-based ensemble learning^18^ excelled in lithofacies classification and permeability prediction for heterogeneous reservoirs. These have significantly improved prediction performance.

Deep learning solves the limitations of traditional methods through automatic feature extraction^19^. It enables end-to-end learning from raw reservoir data eliminating the need for manual feature engineering^20^. CNN models have been applied to predict key reservoir parameters including porosity by capturing local spatial patterns in logging and seismic data^21^. LSTM networks with their ability to handle sequential data have improved predictions in reservoirs with strong vertical continuity^22^. Bidirectional variants like BiLSTM further enhance performance by leveraging both past and future data dependencies^23^. CRNN models combining convolutional and recurrent layers effectively capture both spatial and temporal features in complex carbonate reservoirs^24^. These advancements have made deep learning a powerful tool for reservoir property prediction. Yet existing studies still have obvious gaps most rely on field-measured data for training lacking support from large-scale and diverse synthetic datasets generated by petrophysical models meanwhile single LSTM or BiLSTM cannot fully capture multi-layer features of complex geological data and few studies combine bidirectional sequence modeling with attention mechanisms resulting in insufficient performance in characterizing nonlinear relationships and noise resistance.

To address these limitations this study proposes a petrophysically-driven stacked bidirectional long short-term memory S-BiLSTM neural network with three key novelties. First it generates large-scale diverse synthetic datasets of elastic and anisotropic parameters through shale petrophysical modeling overcoming the constraint of limited field-measured data and providing training data that closely simulates real geological environments. Second it adopts a stacked bidirectional LSTM structure where multiple bidirectional LSTM layers are stacked to capture both forward and backward data dependencies while extracting multi scale features addressing the insufficiency of single LSTM in characterizing complex geological data. Third it integrates an attention mechanism and Dropout regularization to enhance the model’s ability to focus on key features and resist noise ensuring stable performance even with noisy data. Derived from BiLSTM an improved LSTM variant^25^ that realizes bidirectional information propagation through two opposite-direction hidden layers integrating complete past and future information for each sequence point^26^. This S-BiLSTM model is validated using real reservoir parameter data from an oil field in Southwest China. Results confirm its high accuracy robustness and noise immunity in porosity distribution prediction.

## Materials and methods

### Petrophysical modeling

Petrophysical modeling describes subsurface rocks and their properties using mathematical and physical equations. It first calculates key rock properties including acoustic velocities densities normal weakness and tangential weakness from well log data^27^. These calculated properties then serve as inputs for seismic forward modeling which predicts seismic data and relevant reservoir physical parameters by simulating the physical response of different rock types and saturations in the reservoir.

**Fig. 1:**
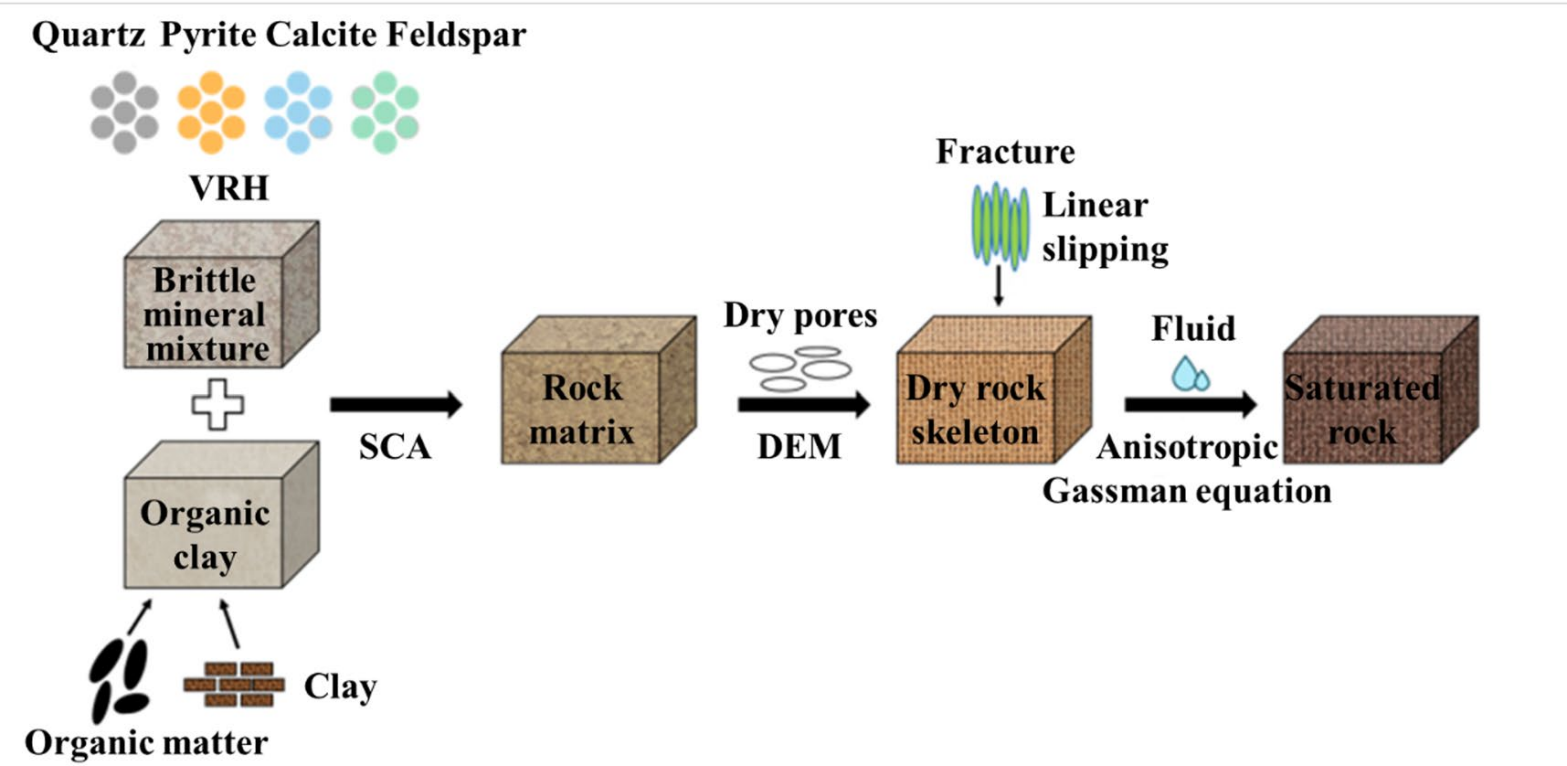
Shale petrophysical modeling process.

Shale oil and gas have garnered significant attention in recent years. To accurately characterize the physical properties of shale rocks, this study constructs an equivalent medium petrophysical model of shale based on multi-theoretical coupling (in Fig. 1). This shale petrophysical model sequentially calculates the matrix modulus dry rock skeleton modulus and fluid-saturated rock modulus by integrating the Voigt-Reuss-Hill averaging method the equivalent medium model the linear slip model and the anisotropic Gassmann fluid substitution equation. It comprehensively accounts for the effects of matrix minerals pore structure oriented fractures and pore fluids in shale to determine the equivalent medium rock modulus and elastic parameters of shale. This petrophysical model provides a comprehensive dataset and a near-actual data environment for training deep learning networks. Training with samples derived from this petrophysical modeling offers a theoretical foundation for the accurate prediction of physical parameters and significantly enhances the generalization of predictions. Subsequently, five parameters - longitudinal wave velocity ($\:{V}_{p}$), transverse wave velocity ($\:{V}_{s}$), rock density ($\:\rho\:$), normal weakness ($\:{\varDelta\:}_{N}$), and tangential weakness ($\:{\varDelta\:}_{T}$) - were selected as inputs to the neural network for predicting porosity. The five parameters were selected for their inherent links to porosity in petrophysical modeling. $\:{V}_{p}$, $\:{V}_{s}$ and $\:\rho\:$ reflect matrix–fluid interactions while $\:{\varDelta\:}_{N}$ and $\:{\varDelta\:}_{T}$ capture fracture-induced elastic anisotropy.

### Long short-term memory neural network

**Fig. 2:**
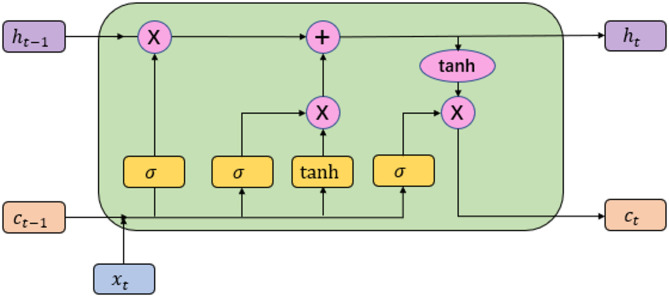
Structure of LSTM neural network.

Long Short-Term Memory networks are an advanced recurrent neural network architecture to address gradient explosion or vanishing and long-term temporal dependency issues of conventional RNNs^28^. As illustrated in Fig. 2, their architecture includes three regulatory gate mechanisms and a dedicated memory cell unit^29^. The memory unit stores input information, the forgetting gate manages information retention or discarding^30^,the input gate updates the memory unit via Sigmoid and tanh functions and the output gate controls information output.

### Stacked bidirectional LSTM neural network modeling

To enhance the predictive capability of traditional LSTM neural networks for reservoir physical property parameters, this paper innovatively proposes a new neural network structure. Key modifications distinct from standard LSTM and BiLSTM include three critical improvements. First, the input sequence direction is extended from unidirectional to bidirectional enabling capture of both forward and backward data dependencies^31^. Second, multiple bidirectional LSTM layers are stacked to extract multi scale features addressing limitations of single layer designs in characterizing complex geological data. Third, an attention mechanism is integrated to prioritize key features alongside Dropout regularization to resist noise. These modifications collectively strengthen feature extraction and noise tolerance critical for reservoir porosity prediction under complex geological conditions. In conclusion, stacked bi-directional LSTM neural network is stronger than traditional LSTM neural network with the following three advantages:

1. Better feature extraction ability: by stacking multiple LSTM layers to extract richer features, can better solve complex tasks;
2. Better capture of long-term dependencies: can capture forward and backward dependencies, can better handle long sequence data;
3. With stronger robustness: through multiple LSTM layers to enhance the resistance to noise and interference, can work in complex environments.

#### Bidirectional LSTM neural network

**Fig. 3:**
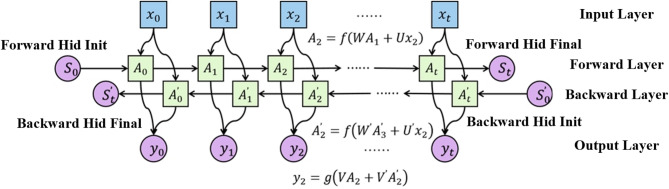
Structure of bidirectional LSTM network (Bi-LSTM).

The structure of the Bi-LSTM neural network is shown in Fig. 3, which combines two sub-networks, the forward and backward LSTMs, and is capable of capturing bidirectional dependencies. Compared with unidirectional LSTM, it can refer to both past and future information at any moment t, and the prediction results are more accurate, which is especially suitable for the task that needs to understand the long-term dependency of elements^32^.

In Fig. 3, $\:W$ and $\:{W}^{{\prime\:}}$ are weight matrices from the input layer to the hidden layer. $\:W$ is used for the forward layer, while $\:{W}^{{\prime\:}}$ is used for the backward layer. They are respectively used to transform the input $\:{x}_{t}$ into the forward hidden state and the backward hidden state. $\:U$ and $\:{U}^{{\prime\:}}$ are weight matrices from the hidden state to the hidden state, used to capture the dependencies between time steps in the sequence. $\:U$ is used for the forward layer, and $\:{U}^{{\prime\:}}$ is used for the backward layer. They are respectively used to compute the recursive relationships of the forward and backward hidden states. $\:{S}_{0}$ is Forward Hid Init and $\:{S}_{t}$ is Forward Hid Final. They are the initial and final state vectors of the forward hidden layer respectively. $\:{S}_{0}^{{\prime\:}}$ is Backward Hid Init and $\:{S}_{t}^{{\prime\:}}$ is Backward Hid Final. They are the initial and final state vectors of the backward hidden layer respectively. $\:{A}_{t}$ and $\:{A}_{t}^{{\prime\:}}$ represent the hidden states of the forward and backward layers at time step $\:t$, respectively. The forward-computing LSTM is labeled as $\:{A}_{0}\to\:{A}_{1}\to\:{A}_{2}\to\:\cdots\:\to\:{A}_{t}$, and its input at the moment t consists of two parts: the sequence data $\:{x}_{t}$ at the current moment and the output $\:{A}_{t-1}$ of $\:\left(t-1\right)$ at the previous moment. The reverse-computing LSTM is labeled as $\:{A}_{0}^{{\prime\:}}\to\:{A}_{1}^{{\prime\:}}\to\:{A}_{2}^{{\prime\:}}\to\:\cdots\:\to\:{A}_{t}^{{\prime\:}}$, and its input at the moment t consists of the sequence data $\:{x}_{t}$ at the current moment and the output $\:{A}_{t+1}^{{\prime\:}}$ of $\:\left(t+1\right)$ at the latter moment. Eventually, the output value is jointly determined by $\:{A}_{t-1}$ and $\:{A}_{t+1}^{{\prime\:}}$, which reflects the advantage of the bidirectional LSTM in processing sequence data that can consider both the preceding and following information. Eventually, the output value is jointly determined by the forward and backward hidden states, as shown by $\:{y}_{2}=g\left(V{A}_{2}+{V}^{{\prime\:}}{A}_{2}^{{\prime\:}}\right)$. This reflects the advantage of the bidirectional LSTM that it can consider both preceding and following information when processing sequence data.

#### Stacked neural network

Stacked neural networks vertically integrate multiple neural architectures into a hierarchical framework that progressively enhances feature extraction capabilities. By sequentially propagating outputs from preceding layers as inputs to subsequent layers through cascaded network tiers, this architecture enables the construction of deep hierarchical representations with superior nonlinear modeling capacity^33^. In the studied, southwest China oilfield the Longmaxi Formation shale reservoir exhibits distinct geological characteristics including deep burial complex pore networks vertical lithological layering and fracture induced lateral heterogeneity. These features create multi scale relationships between elastic parameters and porosity that single layer networks cannot fully capture. Stacked bidirectional LSTM layers address this by capturing basic correlations between elastic parameters and porosity through initial processing while subsequent layers focus on higher order interactions including the combined effects of fracture-induced anisotropy parameters on porosity distribution. This hierarchical division mirrors the reservoir’s nested structure where small scale pores within shale matrix interact with large scale fracture zones to control overall porosity patterns.

#### Parameter settings

In this study, the stacked bidirectional LSTM architecture includes three bidirectional LSTM layers each with 128 neurons. Each bidirectional layer connects forward and backward hidden states to capture sequential dependencies in both directions. An attention layer follows the stacked LSTM layers to emphasize critical features. A dropout layer with a rate of 0.5 is applied between layers to reduce overfitting. The output layer is a fully connected layer with linear activation to produce porosity predictions.

Comparative analysis of activation functions reveals that the ReLU function exhibits superior computational efficiency relative to tanh and sigmoid functions, while simultaneously introducing beneficial sparsity through selective neuron deactivation. This sparsity induction mechanism reduces parameter interdependency by strategically nullifying non-critical neural pathways, thereby optimizing network resource allocation^34^. Therefore, in this paper, when constructing the stacked bidirectional LSTM neural network, the neurons in the hidden layer choose ReLU, and the output layer uses linear activation function, which ensures that the output can take any value.

In the process of neural network training, the main role of the loss function is to quantify the gap between the predicted value $\:f\left(x\right)$ and the actual value y of the network model^35^. The prediction of physical parameters of the reservoir in this paper is essentially a regression prediction problem, so the loss function-mean-square error (MSE) function suitable for regression problems is used, which is given by:

1 $$\:L\left(y,f\left(x\right)\right)=\frac{1}{n}\sum\:_{i=1}^{n}{\left({y}_{i}-f\left({x}_{i}\right)\right)}^{2}$$

In Eq. (1), $\:f\left({x}_{i}\right)$ is the predicted value, $\:{y}_{i}$ is the true value in the training sample, and n is the number of samples trained^36^.

Optimizers play a pivotal role in neural network training by computing and updating network parameters, with appropriate selection critically influencing model convergence speed and overfitting mitigation^37^. Adam was adopted as the optimizer for S-BiLSTM training because among prevalent optimizers it stands out by adaptively adjusting learning rates through gradient variance estimation. This mechanism enables aggressive initial step sizes for rapid early-stage convergence followed by automatic step size reduction for precise late-stage optimization effectively preventing gradient explosion/vanishing while maintaining consistent performance across training phases^38^.

The attention mechanism emulates human cognitive prioritization by dynamically allocating computational resources through weighted feature emphasis, where higher-weight features receive augmented processing focus^39^. Integration of this mechanism within bidirectional LSTM architectures enables enhanced utilization of discriminative data patterns, significantly improving model efficacy.

#### Stacked bidirectional LSTM

**Fig. 4:**
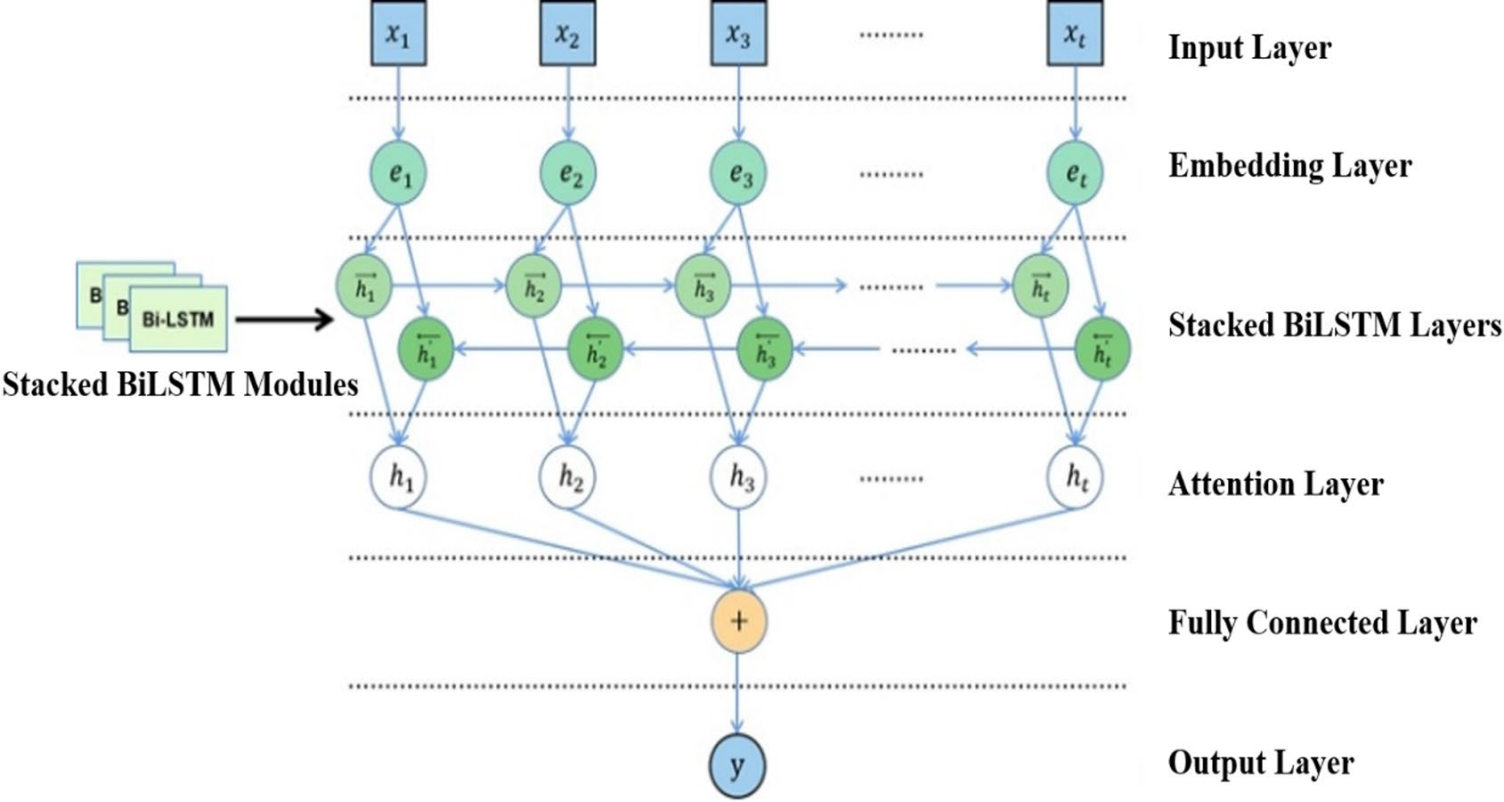
Workflow of the neural network with the attention mechanism.

As demonstrated in Fig. 4, our augmented Stacked Bidirectional LSTM (S-BiLSTM) architecture incorporates attention layers to optimize feature relevance assessment.

The Input Layer accepts the input data and converts it into embedding vectors, which are then converted into dense vectors. The similarity of the training dataset is computed by Stacked BiLSTM layers, and the softmax algorithm is used to assign probabilities in order to obtain the contribution of the hidden layer, which is weighted and summed to obtain the output value. Adding the attention mechanism can improve the generalization performance of S-BiLSTM model^39^.

The workflow of the neural network with the new structure is that when the input sequence, $\:\left({x}_{1},{x}_{2},{x}_{3},\cdots\:,{x}_{t}\right)$ is processed through Stacked BiLSTM Layers that generates two sets of hidden states, $\:\left({h}_{1},{h}_{2},{h}_{3},\cdots\:,{h}_{t}\right)$ and $\:\left({h}_{1}^{{\prime\:}},{h}_{2}^{{\prime\:}},{h}_{3}^{{\prime\:}},\cdots\:,{h}_{t}^{{\prime\:}}\right)$. These two sets of hidden states are fed into the Attention Layer, which integrates them into a context vector that is placed into the Fully Connected Layer, which ultimately outputs the desired value, $\:y$. The neural network’s depth and complexity are greatly increased. Where $\:{e}_{1},\:{e}_{2},\:{e}_{3},\:\cdots\:,\:{e}_{t}$ are the outputs of the Embedding Layer for each time step $\:{x}_{1},\:{x}_{2},\:{x}_{3},\:\cdots\:,\:{x}_{t}$ in the Input Layer. $\:\overrightarrow{{h}_{1}},\:\overrightarrow{{h}_{2}},\:\overrightarrow{{h}_{3}},\:\cdots\:,\:\overrightarrow{{h}_{t}}$ is the hidden state of the forward LSTM layer at each time step. $\:\overleftarrow{{h}_{1}^{{\prime\:}}},\:\overleftarrow{{h}_{2}^{{\prime\:}}},\:\overleftarrow{{h}_{3}^{{\prime\:}}},\:\cdots\:,\:\overleftarrow{{h}_{t}^{{\prime\:}}}$ is the hidden state of the backward LSTM layer at each time step. In the attention layer, $\:{h}_{1},\:{h}_{2},\:{h}_{3},\:\cdots\:,\:{h}_{t}$ represents the hidden state after weighting by the attention mechanism.

### Model evaluation metrics and performance optimization

The optimization of hyperparameters plays a pivotal role in determining neural network performance. For S-BiLSTM architectures, two critical hyperparameters require systematic tuning: hidden layer neuron dimensionality, governing model capacity; and dropout rate, controlling regularization intensity. This paper adopts a progressive optimization strategy that sequentially adjusts these parameters - first establishing network architecture through neuron configuration, and fine-tuning generalization performance through dropout rate modulation. This parameter-decoupled optimization strategy simultaneously enhances computational efficiency and model performance.

#### Model performance evaluation metrics

In the regression prediction task, the model prediction results and the real value can be plotted in the same graph to directly observe the degree of agreement, the image method is concise and intuitive, but is susceptible to subjective influence and lack of specific comparison. Therefore, in addition to the image comparison, this paper also uses the mean absolute error (MAE) and root mean square error (RMSE) to objectively evaluate the model prediction performance.

The formula is as follows:

2 $$\:MAE=\frac{1}{n}\sum\:_{i=1}^{n}\left|{y}_{i}-{\widehat{y}}_{i}\right|$$

3 $$\:RMSE=\sqrt{\frac{1}{n}\sum\:_{i=1}^{n}{\left({y}_{i}-{\widehat{y}}_{i}\right)}^{2}}$$

Where $\:{y}_{i}$ represents the true value of the ith sample point, $\:{\widehat{y}}_{i}$ is the predicted value of the ith sample point, and $\:n$ is the total number of samples.

MAE is less sensitive to outliers, and a smaller MAE value indicates a smaller prediction error. RMSE measures the degree of error between the predicted value and the actual value, the smaller it is, the closer the predicted value is to the actual value, and the higher the prediction accuracy and effect of the model.

#### Hidden neuron number optimization

The number of neurons in the hidden layer is a key factor affecting the complexity of the model, the more the number, the stronger the model expression ability, but the higher the risk of overfitting, according to the size and complexity of the training data to choose the appropriate value^40^.

**Table 1:** Effect of the number of neurons in the hidden layer on the S-BiLSTM model.

Number of neurons in the hidden layer	MAE	RMSE	Predictive accuracy (%)
32	0.0138	0.0275	62.19
64	0.0121	0.0257	67.34
96	0.0097	0.023	73.76
112	0.0073	0.0201	80.04
120	0.0048	0.0163	86.88
124	0.0042	0.0151	88.49
126	0.0031	0.013	91.51
128	0.0027	0.0121	92.75
130	0.0028	0.0123	92.38
132	0.0030	0.0128	92.1
136	0.0031	0.0132	91.49
144	0.0032	0.0133	91.06
160	0.0058	0.0179	84.31
256	0.0073	0.0201	80.27
512	0.008	0.021	78.12

**Fig. 5:**
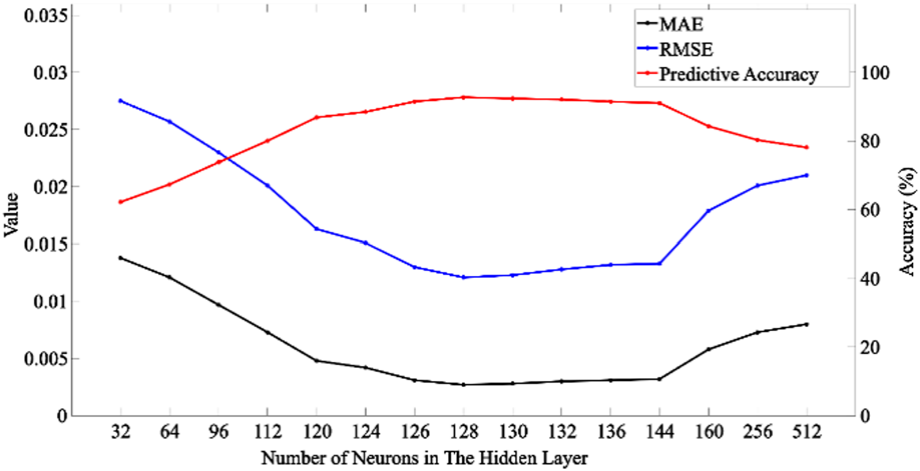
Relationship between the number of neurons in the hidden layer and evaluation indexes.

In the training, the number of hidden layer neurons is set to 32, 64, 128, 256, 512 and iterated for 1000 rounds of test training, and it is found that with the increase of the number, the model’s MAE and RMSE of the physical parameters of the storage layer are high and then low and then rise, and the prediction accuracy is first low and then high and then low, and reaches the local optimal solution at 128. In order to verify whether 128 is the global optimal solution, the hidden layer neurons are set to 96, 112, 120, 124, 126, 130, 132, 136, 144, 160 experiments, and the results are shown in Table 1; Fig. 5, when the number of 128, the S-BiLSTM neural network model has the optimal performance and the highest prediction accuracy. This reflects a trade-off between model capacity and overfitting risk. Increasing the number from 32 to 128 enhances feature extraction ability, which is indicated by decreasing MAE and RMSE values along with rising predictive accuracy. However, exceeding this value leads to performance degradation as a result of overfitting.

#### Dropout size optimization

**Table 2:** Effect of dropout size on the S-BiLSTM model.

Dropout size	MAE	RMSE	Predictive accuracy (%)
0.1	0.0289	0.0399	21.41
0.2	0.0264	0.0381	27.81
0.3	0.0181	0.0315	50.72
0.4	0.0064	0.0189	82.44
0.5	0.0026	0.012	92.75
0.6	0.0035	0.0141	90.51
0.7	0.0053	0.0172	85.39
0.8	0.0097	0.0232	73.67

**Fig. 6:**
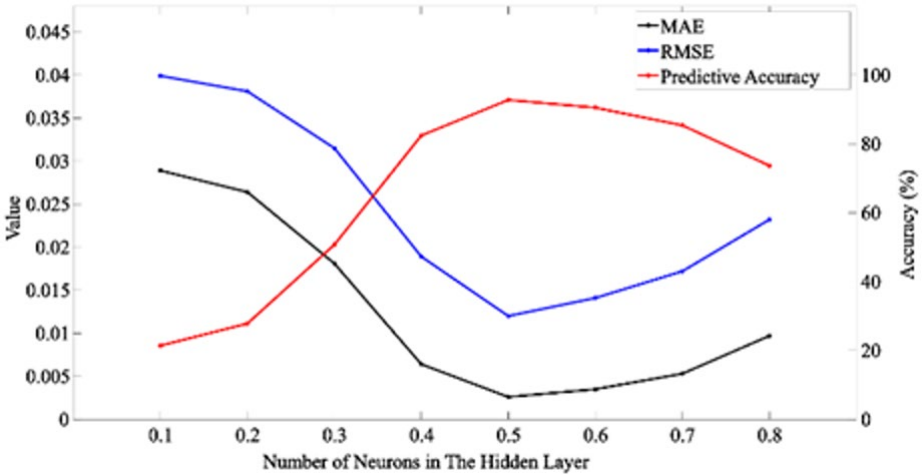
Relationship between dropout size and evaluation indexes.

Determining the optimal Dropout rate requires consideration of several factors such as dataset characteristics, network architecture and parameters during training. In the neural network training, the Dropout size was set to 0.1, 0.2, 0.3, 0.4, 0.5, 0.6, 0.7, 0.8 and 1000 rounds of iterations were performed for test training. The results show that the validation set model is not overfitted and has the highest prediction accuracy when the Dropout size is 0.5.

As shown in Table 2; Fig. 6, different Dropout rates lead to variations in MAE, RMSE, and prediction accuracy. The S-BiLSTM model performs best and achieves the highest prediction accuracy with a Dropout rate of 0.5. This value balances regularization and information retention. In contrast, lower dropout values such as 0.1 to 0.3 inadequately prevent overfitting and result in poorer accuracy, while higher values around 0.7 to 0.8 excessively restrict feature learning and reduce performance.

#### S-BiLSTM model hyperparameter setting

By designing the network structure and optimizing the hyperparameters of this model, the parameter values are set as shown in Table 3. The optimal hyperparameters for the RNN model and the LSTM model were also determined through multiple experiments by setting their batch size to 64 and learning rate to 0.001.

**Table 3:** S-BiLSTM model hyperparameter value settings.

Model hyperparameters	Hyperparameter value
Number of neurons in hidden layer	128
Stacked layers	3
Batch size	256
Learning rate	0.001
Optimizer	Adam
Dropout size	0.5
Activation function	ReLU
Loss function	MSELoss
Iteration number	1000

### Experimental dataset

The experimental data used to train the neural network in this study are based on petrophysical modeling of real logging data from an oil and gas field in southwest China, with 50,000 sets of training data. The field logging data used to build the petrophysical model include details of mineral composition porosity and fracture characteristics of the Longmaxi Formation shale in the study area. These logging data together with pre-stack seismic records of different incident angles provide the basis for generating the synthetic dataset. The longitudinal wave velocity ($\:{V}_{p}$), transverse wave velocity ($\:{V}_{s}$), density ($\:\rho\:$), normal weakness ($\:{\varDelta\:}_{N}$) and tangential weakness ($\:{\varDelta\:}_{T}$) were taken as five parameters as inputs and labeled with the labeled columns of porosity (por). In order to achieve comparison and analysis on a uniform scale, the dataset obtained from the petrophysical model needs to be preprocessed before it is fed into the neural network for training. The data normalization method used in this paper is maximum-minimum normalization, which performs a linear transformation on the sample data to reduce the data range within 0–1^41^.

**Fig. 7:**
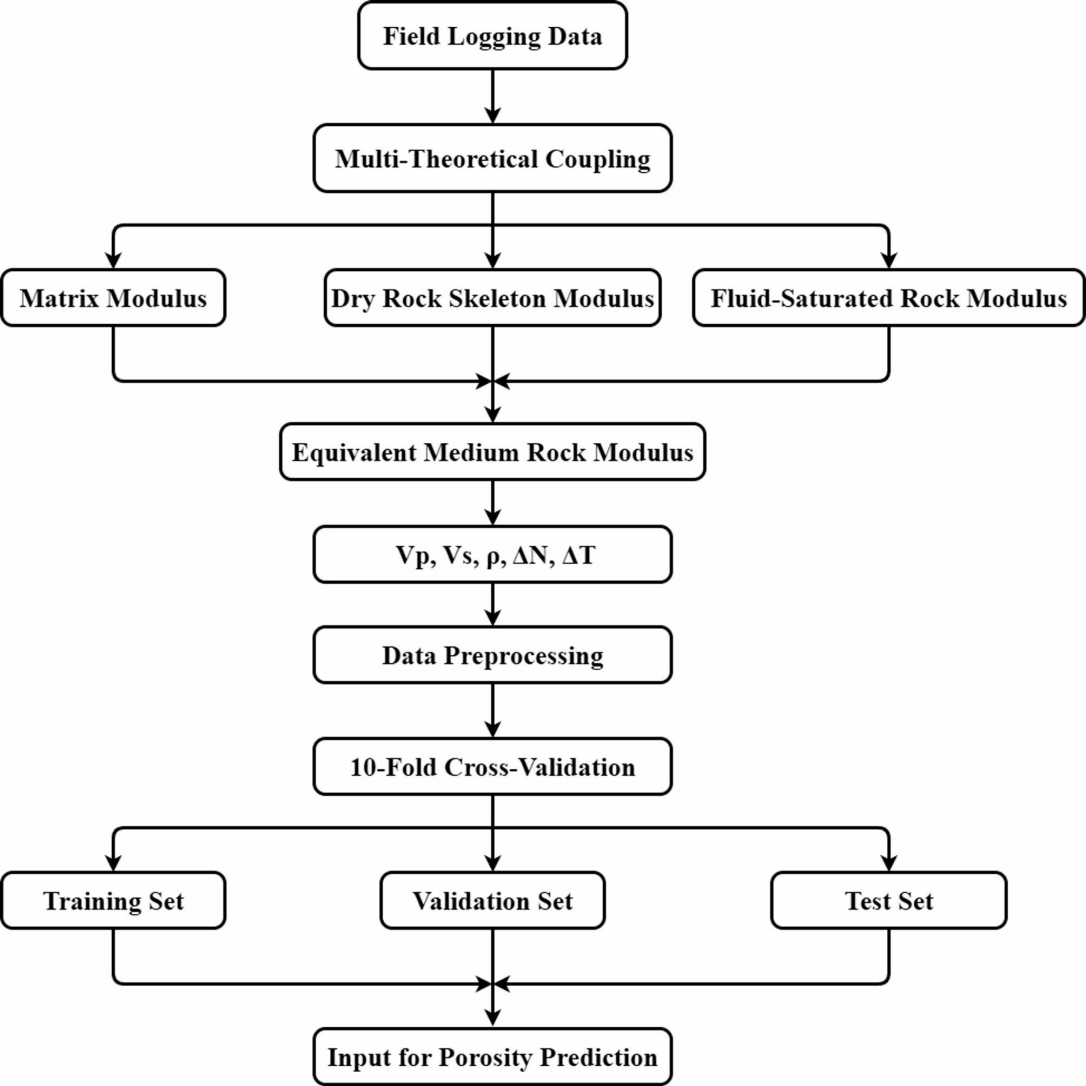
Petrophysical-driven data generation workflow.

The dataset is divided by the cross-validation division method, which can reduce the influence of chance and enhance the model generalization ability and data utilization efficiency, and the ten-fold cross-validation division method is used. The ten-fold cross-validation process begins by randomly dividing the 50000-group dataset into ten equal subsets each with 5000 groups^42^. During each iteration one subset is used as the validation set for evaluation and the other nine form the training set for S-BiLSTM model training^43^. This cycle repeats ten times so every subset serves as the validation set once^44^. Final performance metrics including MAE RMSE and predictive accuracy are determined by averaging all validation outcomes improving robustness and reducing partitioning randomness^45^. The dataset in this study is first divided into training set and test set according to 9:1, and the training set is further divided into training set and validation set according to 9:1, with 81% of the training set (40,500 groups), 9% of the validation set (4,500 groups), and 10% of the test set (5,000 groups). As shown in Fig. 7; Table 4, the entire data generation workflow and final results are presented.

In order to ensure the generalization performance of the model, the training set is used for training and then the validation set is used for secondary validation, and finally the test set is used to objectively assess the prediction effect.

**Table 4:** Table of details of data set segmentation.

Data sources	Data set categories (input features; labeled columns)	Total number of data sets (groups)	Training set (groups)	Validation set (groups)	Test set (groups)	Delineation method
Petrophysical model	$\:{V}_{p}$,$\:{V}_{s}$,$\:\rho\:$,$\:{\varDelta\:}_{N}$, $\:{\varDelta\:}_{T},$ *por*	50,000	40,500	4500	5000	Ten-fold cross validation

## Results

### S-BiLSTM model application performance

After the training of the neural network model was completed, it was analyzed in comparison with RNN, LSTM models and S-BiLSTM model without the Attention layer added to validate the performance of the model in this paper. Porosity prediction was performed separately and model performance was evaluated using mean absolute error (MAE) and root mean square error (RMSE). When dividing the dataset, the impact of the dataset size on the model performance is taken into account, so it is found through experiments that 81% of the training set size is a reasonable choice, which strikes a balance between the model performance and generalization ability.

**Table 5:** Evaluation metrics of different models for porosity prediction.

Neural network model	MAE	RMSE	Predictive accuracy (%)
RNN	0.0059	0.0154	78.13
LSTM	0.0042	0.013	84.38
Un-attention S-BiLSTM	0.0031	0.010	87.50
S-BiLSTM	0.0021	0.0092	92.19

**Table 6:** Comparison of computational efficiency of model training.

Neural network model	Training time (s)
RNN	181.82
LSTM	188.55
Un-attention S-BiLSTM	146.30
S-BiLSTM	161.35

**Fig. 8:**
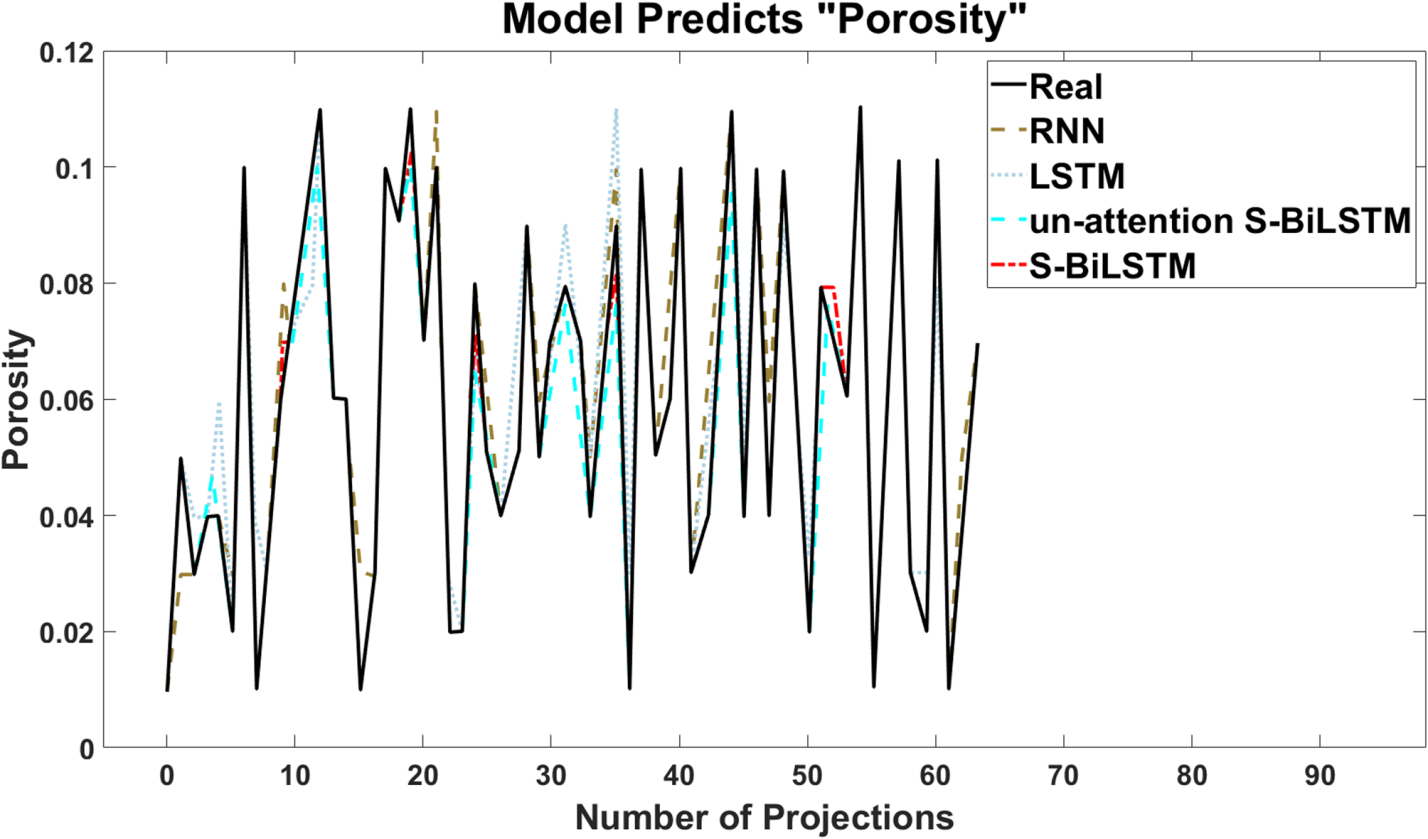
Prediction of “porosity” by each neural network model.

For a better presentation, 64 data points were selected from the model’s 5000-group test set for visual comparison, and the model’s overall performance was evaluated based on the entire 5000-group test set. The advantages and potential of the S-BiLSTM model are more clearly demonstrated using graphical methods. Figure 8 shows a one-dimensional plot of the predicted versus true values of porosity by the four neural networks, and Table 5 summarizes the evaluation data of the four neural networks. Table 6 labels the time spent on the training of each different model, and it can be seen that the S-BiLSTM model is more computationally efficient than RNN and LSTM.

It can be observed that the S-BiLSTM neural network has the best prediction effect, S-BiLSTM neural network without added attention mechanism is next, then LSTM neural network, and lastly RNN neural network. The porosity prediction accuracy of S-BiLSTM model is improved by 7.81% compared with that of the LSTM model, and 14.06% compared with that of the RNN model. And the porosity prediction accuracy is improved by 4.69% after adding the attention mechanism. The reliability of these performance improvements is supported by our ten-fold cross-validation. The S-BiLSTM exhibited lower MAE and RMSE than RNN, LSTM, and the un-attention S-BiLSTM in every validation fold. This consistent error reduction across all data partitions confirms the model’s superiority is not a result of randomness. In the comparison of Fig. 8, the S-BiLSTM neural network also has the best fit between the predicted and real values, which is better than the other networks.

### S-BiLSTM model noise resistance analysis

In the comparison test in the previous section, the trained S-BiLSTM model outperformed the other models in porosity prediction. However, real data are often contaminated by noise, which requires the neural network to maintain high prediction accuracy in the face of noisy data. Real geological signals are preserved through time-depth calibration band-pass filtering and abnormal data detection in data preprocessing. The attention mechanism and Dropout technique in model design help reduce noise interference and avoid artifact generation. In order to verify the noise resistance of the model in this paper, three sets of Gaussian white noise data with signal-noise ratios of 10dB, 5dB and 1dB are set up in the experiments. These SNR levels are selected based on actual seismic data characteristics of the target oilfield in southwest China. The SNR of seismic data in this area generally ranges from 1dB to 10dB. Areas with high well control and low data interference have an SNR of approximately 10dB. Most conventional exploration areas have an SNR of approximately 5dB. Deep complex geological structure areas have an SNR as low as 1dB due to multiple interferences. These settings fully cover the noise scenarios that may be encountered in actual exploration of the study area. MAE, RMSE and prediction accuracy are used as the evaluation indexes to compare the noise resistance performance of the four neural network models RNN, LSTM, Un-attention S-BiLSTM and S-BiLSTM.

**Table 7:** Statistics of model prediction metrics under different signal-noise ratios.

Signal-noise ratio	Neural network model	MAE	RMSE	Predictive accuracy (%)
10dB	RNN	0.0109	0.0209	59.38
	LSTM	0.0083	0.0183	68.75
	Un-attention S-BiLSTM	0.0066	0.0156	76.56
	S-BiLSTM	0.0046	0.0136	82.81
5dB	RNN	0.0124	0.0224	53.75
	LSTM	0.0093	0.0192	64.44
	Un-attention S-BiLSTM	0.0074	0.0169	73.44
	S-BiLSTM	0.0054	0.0149	80.03
1dB	RNN	0.0147	0.0243	44.81
	LSTM	0.0125	0.0224	53.5
	Un-attention S-BiLSTM	0.0089	0.0186	65.63
	S-BiLSTM	0.0063	0.0159	76.12

The performance of the above four models in predicting porosity under different signal-noise ratios was investigated. As shown in Table 7, the S-BiLSTM model performs best at high SNR (10 dB), medium SNR (5 dB), and low SNR (1 dB), and the general trend observed is that the performance of all models deteriorated as the SNR decreased, but the S-BiLSTM network is always in the leading position, followed by the LSTM with the RNN model performing the least effectively. It can also be seen that both noise immunity and prediction performance are significantly improved by adding the attention mechanism. The advantages of S-BiLSTM neural network lie in its stacking structure, bidirectional LSTM structure, Dropout technique and the introduction of attention mechanism. Its stacking structure can learn deep nonlinear relationships. The bidirectional LSTM structure provides a comprehensive understanding of the sequence data, and the Dropout technique reduces the risk of overfitting and enhances the generalization ability. The attention mechanism enables the model to focus on important information. As a result, it can maintain high accuracy with low signal-noise ratio, highlighting its superiority in handling noisy data.

### Practical applications

In this study, the physical parameters of the reservoir are predicted with the help of neural network, based on the relationship between elasticity and physical parameters and the timing characteristics, and the parameters are input into the network by seismic inversion, so as to obtain accurate physical parameter prediction results.

#### Petrophysical modeling and analysis of shale in the actual work area

**Fig. 9:**
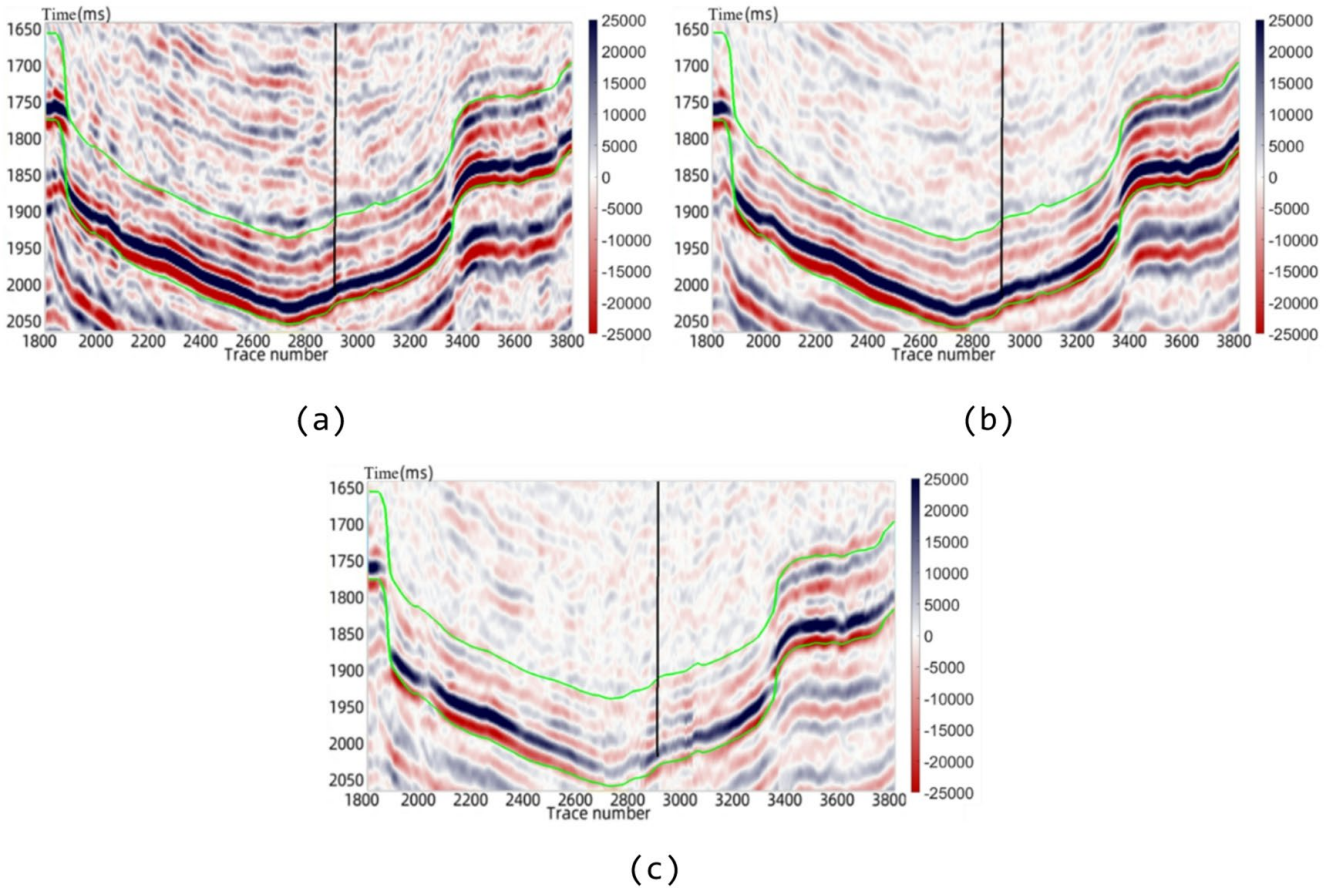
Prediction stacking seismic records with different incidence angles. (**a**) 9°, (**b**) 18°, (**c**) 27°.

In practical applications, taking a work area in southwest China as an example. This work area is located in the north of the southern Sichuan low-fold structural belt in the Sichuan Basin. The NE-trending structure is dominant. From west to east, the Gufoshan structure, Desheng syncline, Longdongping-Jiukuishan anticline, Yunjin syncline and others are developed, presenting the characteristics of wide and gentle synclines and narrow anticlines. The main reservoir is high-quality shale of the Longmaxi Formation. This shale has a large burial depth. The main body is buried at a depth between 3500 and 4000 m. Some are buried at a depth of 4000 to 4500 m, and are scattered at 3000 to 3500 m. The thickness of high-quality shale is 30 to 60 m. Pre-stack seismic records for this area are presented in Fig. 9.

To match the frequency and sampling rate of seismic data, three key measures are implemented. First, time-depth calibration is carried out. The log acoustic travel time curve in the work area is used to establish the depth-time conversion relationship. Elastic and anisotropic parameters including P-wave velocity, S-wave velocity, density, normal weakness and tangential weakness derived from log data are converted from the depth domain to the time domain. The sampling rate of log data in the depth domain is 0.125 m, and the sampling rate of seismic data in the time domain is 2 milliseconds to achieve sampling rate matching. Second, band-pass filtering is applied. Based on the frequency range of elastic parameters derived from log data, band-pass filtering is performed on seismic data to ensure consistent frequency ranges between the two. The frequency range of log-derived parameters is 5–80 Hz, and the frequency range of seismic data is aligned with it after filtering. Third, dynamic correction and gather flattening are conducted. Dynamic correction is used to eliminate time differences between seismic traces caused by differences in propagation paths. Gather flattening aligns signals from the same reflection interface on the time axis to further ensure signal consistency after sampling rate matching.

**Fig. 10:**
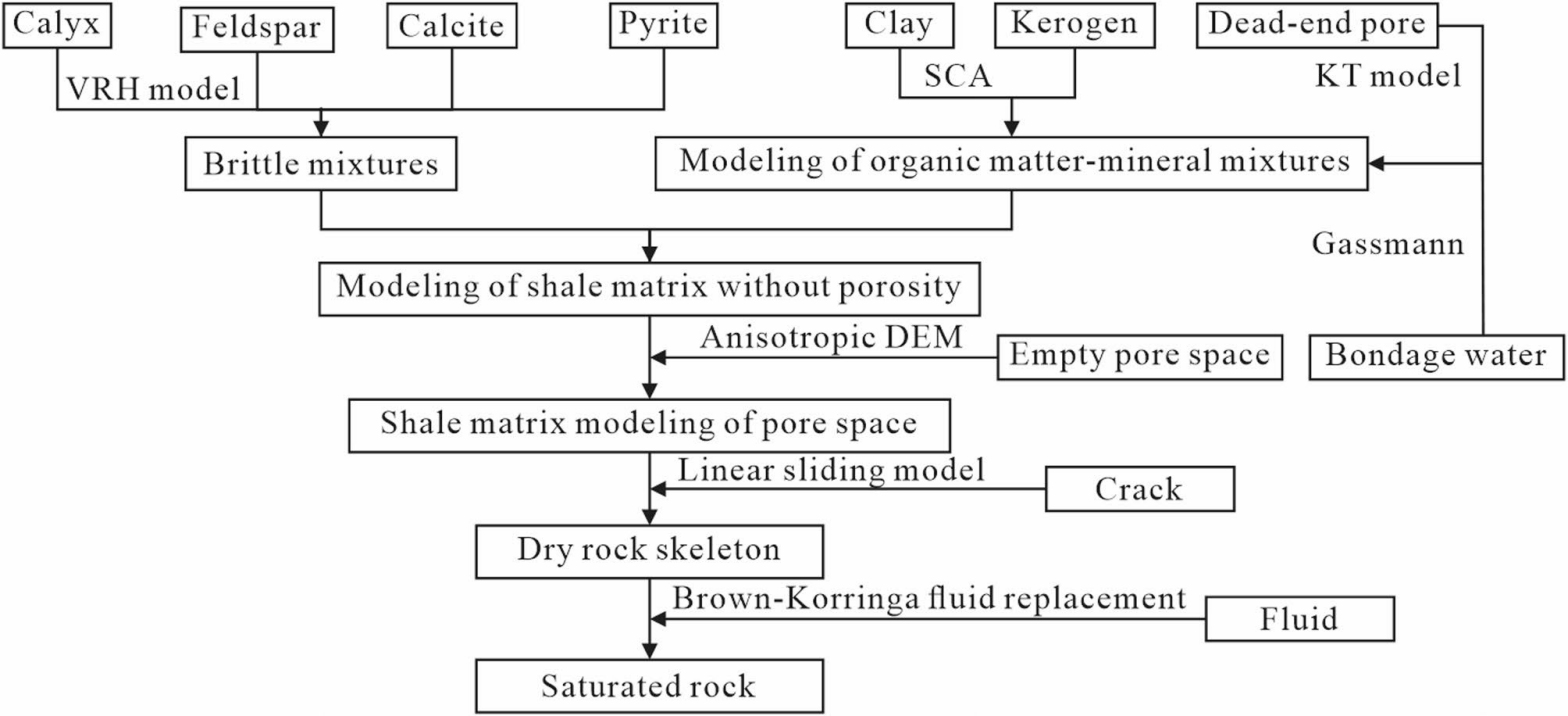
Shale petrophysical modeling process.

The establishment of the petrophysical model provides the necessary training dataset for the subsequent neural network training. The shale petrophysical modeling process is illustrated in Fig. 10. The accuracy and reliability of the model parameters significantly impact the training efficacy of the neural network model.

**Fig. 11:**
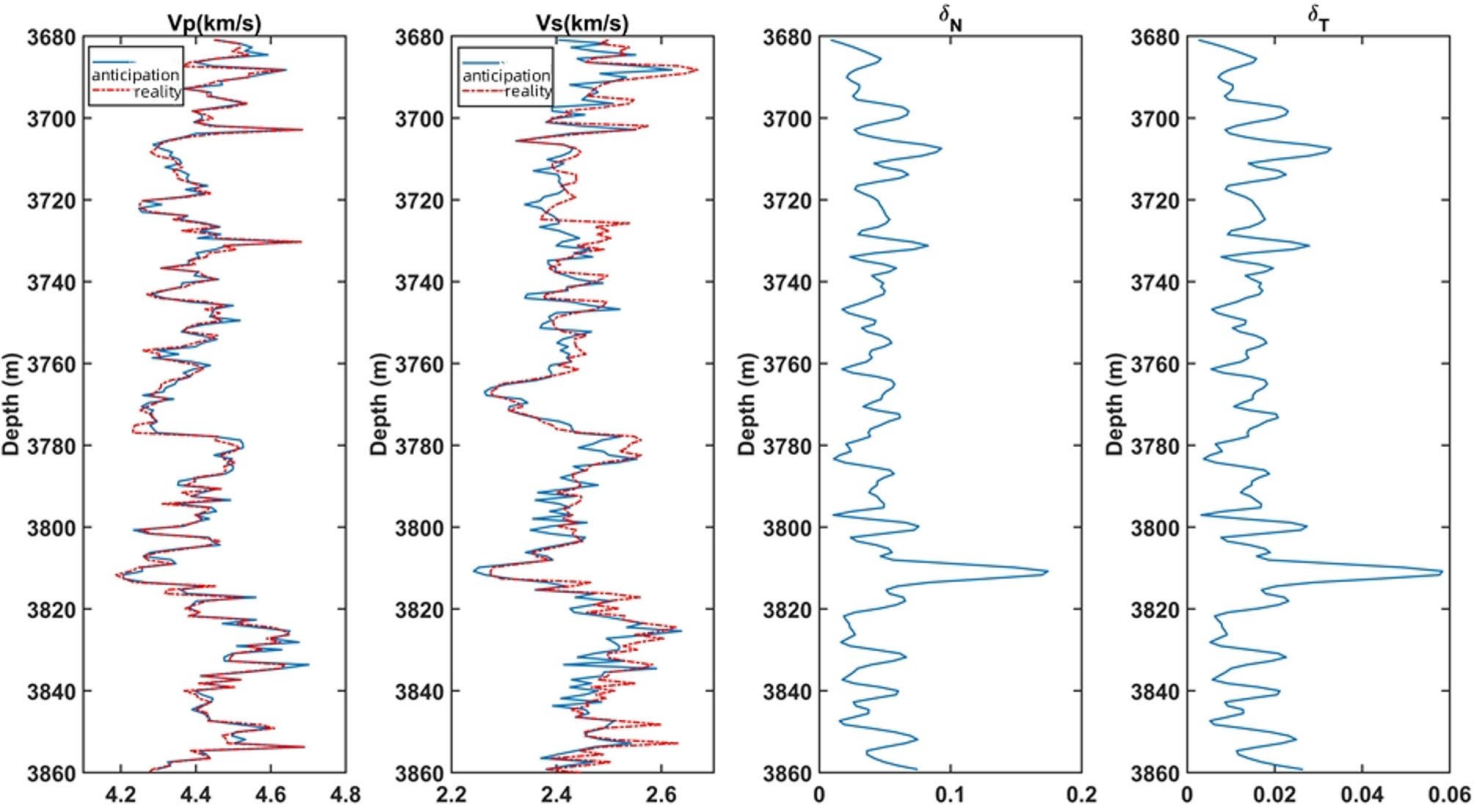
Petrophysical modeling results of the Longmaxi formation shale stratum in a well in the actual work area. (**a**) longitudinal wave velocity; (**b**) transverse wave velocity; (**c**) normal weakness; (**d**) tangential weakness.

Based on the established modeling techniques and processes, the well log interpretation results were employed to accurately predict the fracture and anisotropy parameters along the wellbore, as illustrated in Fig. 11. The average absolute errors for the predicted longitudinal and transverse wave velocities were 1.41% and 1.72%, respectively. These results substantiate the validity and reliability of the model. Figure 11c,d correspond to normal and tangential weaknesses, showing fracture development in some areas.

**Fig. 12:**
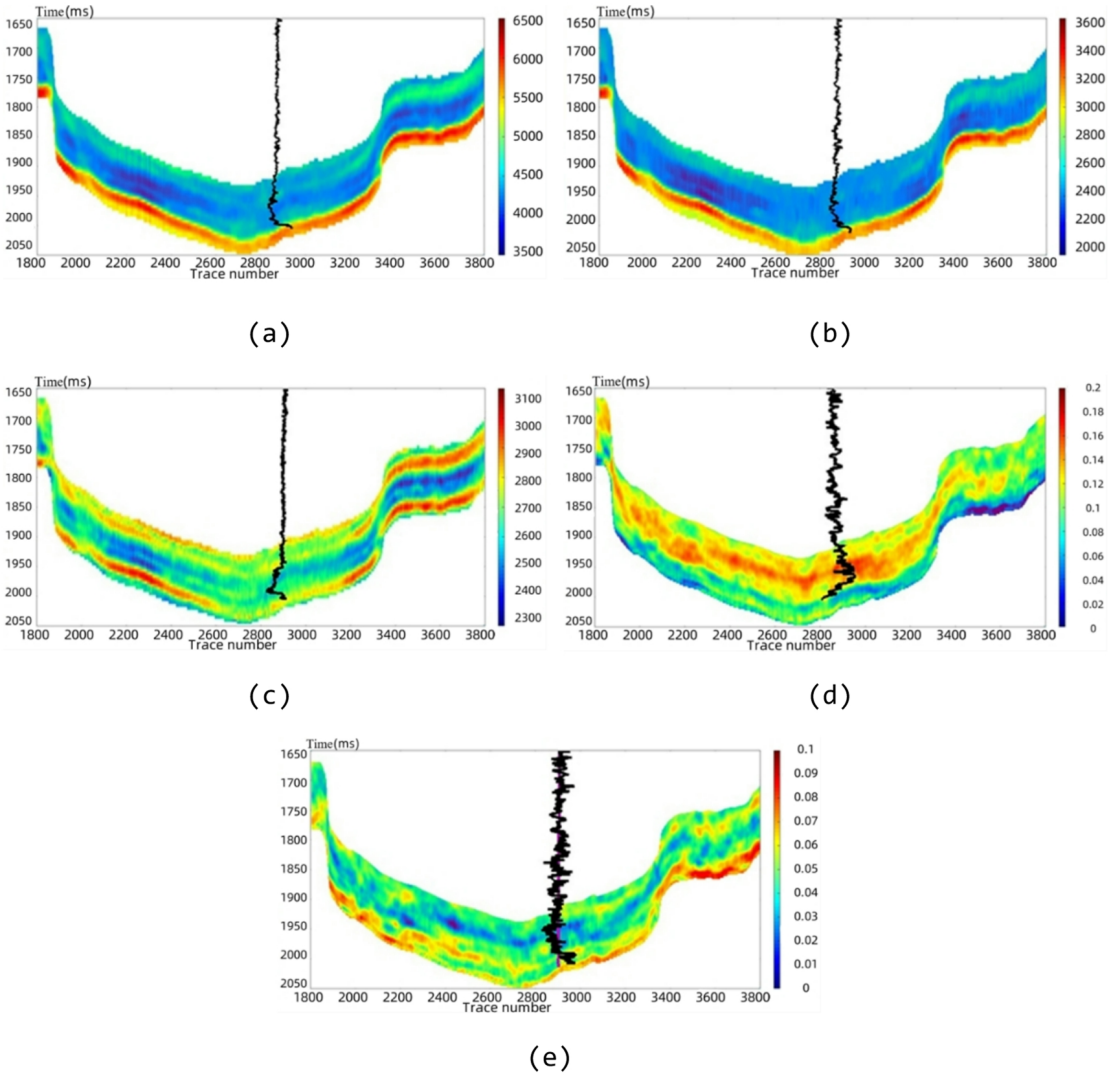
Reservoir profiles for each parameter. (**a**) longitudinal wave velocity, (**b**) shear wave velocity, (**c**) density, (**d**) normal weakness, (**e**) tangential weakness.

Figure 12 shows the results obtained by AVA approximation equations and inversion techniques for the target reservoir parameters in the actual work area. Where (a) and (b) are the longitudinal and transverse wave velocity, (c) is the density, and (d) and (e) are the normal and tangential weakness. From the profile, it can be seen that the density is high in the upper and lower parts of the reservoir and low in the middle, and the cracks are more developed in the area of road number 3500–3800 and depth 1850 m.

#### S-BiLSTM model vs. cross-plot method

**Fig. 13:**
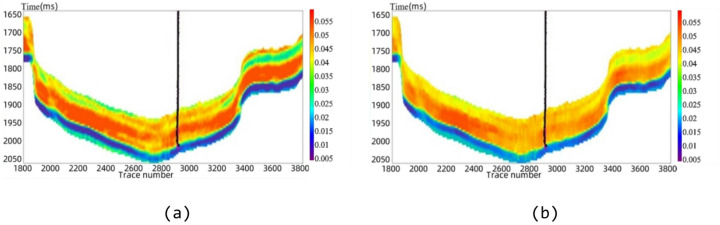
Section of porosity prediction results. (**a**) S-BiLSTM model, (**b**) cross-plot method.

The elasticity and anisotropy parameters inverted in the previous section are inputted into the trained neural network to obtain the predicted physical properties parameters. The cross-plot method establishes a statistical relationship model based on the reservoir parameters and logging profile data, and Fig. 13a shows the prediction results of reservoir porosity by the S-BiLSTM model, and Fig. 13b shows the calculation results of the cross-plot method. It can be seen that in porosity prediction, the S-BiLSTM neural network predicts the profile with continuous and smooth color, which can better capture the subtle changes, while the cross-plot method has discrete color and poor detail capture. Its prediction results are highly consistent with actual geological characteristics. The area with trace numbers 3500–3800 and at a depth of 1850 m in the actual work area is a fracture-developed zone. The high porosity distribution predicted by the S-BiLSTM model in this area is consistent with the fracture characteristics of this zone and also matches the fracture development shown by the imaging logs. Meanwhile, the porosity content of the Longmaxi Formation reservoir in a well of the actual work area varies from 0.73% to 6.8%. The proportion of samples with predicted values of the S-BiLSTM model within this range reaches 92.75%, which further verifies the correctness of the prediction results. The cross-plot method has discrete colors in the predicted profile and shows poor detail capture. The proportion of its predicted values within the above porosity range is only 84.31%. In terms of quantitative metrics, the cross-plot method for porosity prediction yields an MAE of 0.0058, an RMSE of 0.0179, and a predictive accuracy of 84.31%. Here, predictive accuracy is defined as the percentage of samples for which the relative error between the predicted and measured porosity is less than 5%. In contrast, the S-BiLSTM model achieves an MAE of 0.0026, an RMSE of 0.0120, and a predictive accuracy of 92.75%. Compared to the cross-plot method, the S-BiLSTM model reduces the MAE by 0.0032 and the RMSE by 0.0059 while improving predictive accuracy by 8.44%.

In summary, the S-BiLSTM neural network demonstrates significant advantages in predicting reservoir physical properties parameters. The results are more consistent with actual conditions, thereby providing reliable data for reservoir evaluation.

#### S-BiLSTM model vs. support vector machine (SVM)

**Fig. 14:**
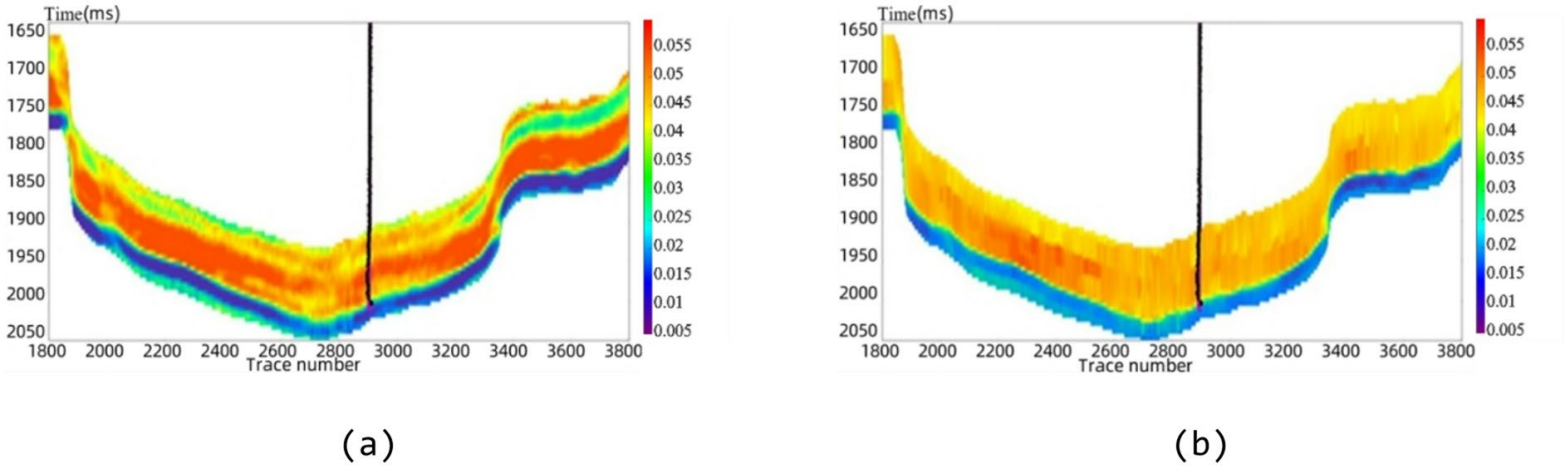
Section of porosity prediction results. (**a**) S-BiLSTM model, (**b**) SVM model.

Figure 14a will show the prediction results of reservoir porosity by S-BiLSTM neural network. Figure 14b, on the other hand, shows the prediction results obtained using Support Vector Machine (SVM).

**Fig. 15:**
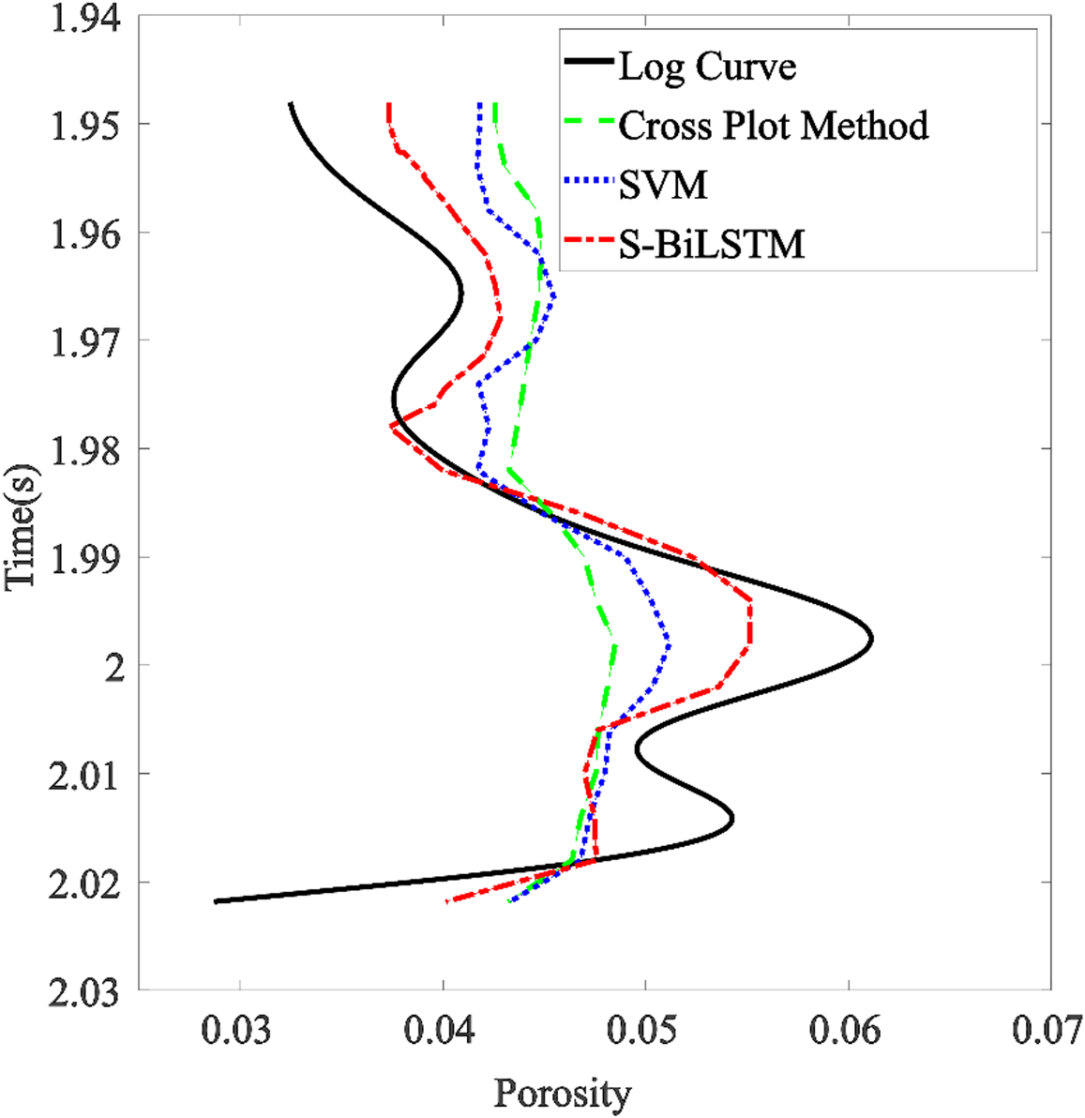
Comparison of porosity prediction and logging curves by methods.

Comparing the profiles and logging curves of the two methods, the S-BiLSTM model predicts finer results than SVM. Furthermore, when juxtaposed with the cross-plot method, the SVM model demonstrates certain advantages. Quantitative comparison shows that the SVM for porosity prediction has an MAE of 0.0042, an RMSE of 0.0146, and a predictive accuracy of 87.38%. The S-BiLSTM model, in contrast, has an MAE of 0.0026, an RMSE of 0.0120, and a predictive accuracy of 92.75%. Compared to the SVM, the S-BiLSTM reduces the MAE by 0.0016 and the RMSE by 0.0026, while improving predictive accuracy by 5.37%. As illustrated in Fig. 15, the comparison of logging curves reveals that the data produced by the S-BiLSTM neural network aligns more closely with the actual logging values than the predicted values obtained from the SVM model. This indicates that the S-BiLSTM model is more effective and superior to the SVM model in predicting the physical parameters of the reservoir. The predictions made by the S-BiLSTM model are accurate and reliable, showcasing significant advantages over the SVM model.

## Discussion

Addressing the prevalent issues of suboptimal prediction accuracy and inadequate noise resistance in porosity prediction, this study introduces a novel framework that integrates stacked bidirectional Long Short-Term Memory (S-BiLSTM) neural networks with petrophysical modeling for enhanced reservoir porosity parameter prediction. Specifically, the incorporation of shale petrophysical modeling facilitates the generation of comprehensive and diverse training datasets, thereby enabling the neural network to effectively simulate the actual data environment and discern intricate relationships within the data.The S-BiLSTM architecture excels in capturing long-term dependencies and contextual information through its multi-layer LSTM network and bidirectional information transfer mechanism. This is complemented by the integration of several advanced techniques, including the ReLU activation function, MSE loss function, Adam optimization algorithm, Dropout regularization, and Attention mechanism, which collectively enhance the model’s nonlinear fitting capabilities and robustness.

For model evaluation and performance optimization, systematic experimentation was conducted to determine optimal hyperparameters, including the number of neurons in the hidden layers, batch size, and Dropout rate. The visualization tools employed in this study provide valuable insights into the model’s learning process and feature interpretation, thereby enhancing its interpretability and explanatory power. In terms of feature importance, the contributions of the five input parameters to porosity prediction align with their petrophysical significance as elaborated in Sect. 2.1. Longitudinal wave velocity, transverse wave velocity, and rock density, which reflect matrix-fluid interactions, play relatively prominent roles in prediction due to their direct association with pore fluid storage capacity. Normal weakness and tangential weakness, which capture fracture-induced elastic anisotropy, contribute to predicting porosity variations in fractured zones, complementing the matrix-related parameters.

Comparative analysis demonstrates that the S-BiLSTM model exhibits superior performance in porosity prediction, achieving higher precision and accuracy relative to conventional RNN and LSTM networks. Practical application of the model in a southwestern Chinese oil and gas field yielded promising results, with the S-BiLSTM predictions demonstrating superior accuracy and reliability when compared against cross-plot analysis, Support Vector Machine (SVM) methods, and actual logging data. Such reliable porosity predictions can further support operational decisions in drilling by helping identify areas with more favorable reservoir conditions to optimize target selection. Beyond prediction accuracy, computational efficiency is another critical factor for engineering application of the model. According to the training time presented in Table 6, the S-BiLSTM model exhibits higher computational efficiency than RNN and LSTM models under the current dataset.

However, its stacked bidirectional LSTM layers and attention mechanism may lead to increased computational costs when handling larger-scale or higher-dimensional datasets. While this framework has demonstrated strong porosity prediction performance with real field data, it is worth noting that the synthetic data generated through petrophysical modeling may not fully capture the differences in petrophysical properties of Longmaxi Formation shale induced by varying burial depths and may further fail to reflect the random micro-fracture distribution observed in actual logging data. A key direction for future research will be to integrate core data from different well depths with synthetic data to optimize the petrophysical model; we will further test the S-BiLSTM model on reservoirs with different lithologies to verify its generalization capability across diverse geological settings and plan to combine this model with real-time logging or seismic data to enhance its applicability in dynamic reservoir evaluation processes. The S-BiLSTM model’s generalization capability stems from its powerful feature learning mechanisms. These mechanisms include the stacked bidirectional LSTM structure and the attention mechanism, which enable the model to capture complex patterns and relationships in data. This advantage further makes the model show promising effectiveness on other datasets. To further enhance this generalization capability, more diverse datasets can be explored, and the model’s architecture and hyperparameters can be continuously optimized based on the features of new data.

## Conclusions

The stacked bidirectional LSTM neural network prediction method based on petrophysical driving proposed in this paper offers a novel approach for predicting reservoir physical property parameters. Validated in the oilfield application in southwest China, it supports operational decisions in drilling by helping identify areas with more favorable reservoir conditions and thus optimizes target selection. The S-BiLSTM model achieves a porosity prediction accuracy of 92.19% which is 7.81% higher than that of the LSTM model and 14.06% higher than that of the RNN model. It maintains high accuracy under noisy data with 82.81% accuracy at a signal-to-noise ratio of 10dB and 76.12% accuracy at a signal-to-noise ratio of 1dB. Compared with the cross-plot method it reduces the mean absolute error by 0.0032 and improves predictive accuracy by 8.44% and compared with the support vector machine it reduces the mean absolute error by 0.0016 and improves predictive accuracy by 5.37%. It demonstrates good applicability and also shows broad prospects.

## Data Availability

The datasets used and/or analyzed during the current study available from the corresponding author on reason able request.

## References

[1] Vukadin, D., Čogelja, Z., Vidaček, R. & Brkić, V. Lithology and porosity distribution of High-Porosity sandstone reservoir in North Adriatic using machine learning synthetic well catalogue. *Appl. Sci.* **13**, 7671 (2023).

[2] Bom, C. R. et al. Bayesian deep networks for absolute permeability and porosity uncertainty prediction from image borehole logs from Brazilian carbonate reservoirs. *J. Petrol. Sci. Eng.* **201**, 108361 (2021).

[3] Yang, Y., Wang, D., Yang, J., Wang, B. & Liu, T. Fractal analysis of CT images of tight sandstone with anisotropy and permeability prediction. *J. Petrol. Sci. Eng.* **205**, 108919 (2021).

[4] Khaksar, A. & Griffiths, C. M. *Porosity form Sonic log in gas-bearing Shaly sandstones: field data versus empirical equations. *Explor. Geophys.* **29**, 440–446 (1998).

[5] Mohaghegh, S., Arefi, R., Ameri, S., Aminiand, K. & Nutter, R. Petroleum reservoir characterization with the aid of artificial neural networks. *J. Petrol. Sci. Eng.* **16**, 263–274 (1996).

[6] Ao, Y., Lu, W., Hou, Q. & Jiang, B. Sequence-to-sequence borehole formation property prediction via multi-task deep networks with sparse core calibration. *J. Petrol. Sci. Eng.* **208**, 109637 (2022).

[7] Olutoki, J. O. et al. Estimating petrophysical properties using Geostatistical inversion and data-driven extreme gradient boosting: A case study of late eocene McKee formation, Taranaki Basin, new Zealand. *Results Eng.* **24**, 103494 (2024).

[8] Mohammad Hossain, T., Hermana, M. & Oluwadamilola Olutoki, J. Porosity prediction and uncertainty Estimation in tight sandstone reservoir using Non-Deterministic XGBoost. *IEEE Access.* **12**, 139358–139367 (2024).

[9] Zou, C., Zhao, L., Xu, M., Chen, Y. & Geng, J. Porosity prediction with uncertainty quantification from multiple seismic attributes using random forest. *JGR Solid Earth*. **126**, e2021JB021826 (2021).

[10] Anifowose, F., Labadin, J. & Abdulraheem, A. Improving the prediction of petroleum reservoir characterization with a stacked generalization ensemble model of support vector machines. *Appl. Soft Comput.* **26**, 483–496 (2015).

[11] Jiang, D. et al. A new method for dynamic predicting porosity and permeability of low permeability and tight reservoir under effective overburden pressure based on BP neural network. *Geoenergy Sci. Eng.* **226**, 211721 (2023).

[12] Leisi, A. & Saberi, M. R. Petrophysical parameters Estimation of a reservoir using integration of wells and seismic data: a sandstone case study. *Earth Sci. Inf.* **16**, 637–652 (2023).

[13] Zhang, Z., Wang, Y. & Wang, P. On a deep learning method of estimating reservoir porosity. *Math. Probl. Eng.* **2021**, 1–13 (2021).

[14] Akande, K. O., Owolabi, T. O., Olatunji, S. O. & AbdulRaheem, A. A hybrid particle swarm optimization and support vector regression model for modelling permeability prediction of hydrocarbon reservoir. *J. Petrol. Sci. Eng.* **150**, 43–53 (2017).

[15] Abbas, M. A., Al-Mudhafar, W. J. & Wood, D. A. Improving permeability prediction in carbonate reservoirs through gradient boosting hyperparameter tuning. *Earth Sci. Inf.* **16**, 3417–3432 (2023).

[16] Zanganeh Kamali, M. et al. Permeability prediction of heterogeneous carbonate gas condensate reservoirs applying group method of data handling. *Mar. Pet. Geol.* **139**, 105597 (2022).

[17] Anifowose, F., Abdulraheem, A. & Al-Shuhail, A. A parametric study of machine learning techniques in petroleum reservoir permeability prediction by integrating seismic attributes and wireline data. *J. Petrol. Sci. Eng.* **176**, 762–774 (2019).

[18] Al-Mudhafar, W. J. & Wood, D. A. Tree-based ensemble algorithms for lithofacies classification and permeability prediction in heterogeneous carbonate reservoirs. In *Offshore Technology Conference, OTC*10.4043/31780-MS (Offshore Technology Conference, 2022).

[19] Hinton, G. E., Osindero, S. & Teh, Y. W. A fast learning algorithm for deep belief Nets. *Neural Comput.* **18**, 1527–1554 (2006). 16764513 10.1162/neco.2006.18.7.1527

[20] Zhu, L., Wei, J., Wu, S., Zhou, X. & Sun, J. Application of unlabelled big data and deep semi-supervised learning to significantly improve the logging interpretation accuracy for deep-sea gas hydrate-bearing sediment reservoirs. *Energy Rep.* **8**, 2947–2963 (2022).

[21] Graczyk, K. M. & Matyka, M. Predicting porosity, permeability, and tortuosity of porous media from images by deep learning. *Sci. Rep.* **10**, 21488 (2020). 33293546 10.1038/s41598-020-78415-x PMC7722859

[22] Hochreiter, S. & Schmidhuber, J. Long Short-Term memory. *Neural Comput.* **9**, 1735–1780 (1997). 9377276 10.1162/neco.1997.9.8.1735

[23] Chen, W., Yang, L., Zha, B., Zhang, M. & Chen, Y. Deep learning reservoir porosity prediction based on multilayer long short-term memory network. *GEOPHYSICS* **85**, WA213–WA225 (2020).

[24] Mehrabi, A., Bagheri, M., Bidhendi, M. N., Delijani, E. B. & Behnoud, M. Improved porosity Estimation in complex carbonate reservoirs using hybrid CRNN deep learning model. *Earth Sci. Inf.* **17**, 4773–4790 (2024).

[25] Graves, A. & Schmidhuber, J. Framewise phoneme classification with bidirectional LSTM and other neural network architectures. *Neural Netw.* **18**, 602–610 (2005). 16112549 10.1016/j.neunet.2005.06.042

[26] Jia, H., Yang, Y., An, J. & Fu, R. A. Ship trajectory prediction model based on Attention-BILSTM optimized by the Whale optimization algorithm. *Appl. Sci.* **13**, 4907 (2023).

[27] Fu, L. Joint inversion of seismic data for acoustic impedance. *GEOPHYSICS* **69**, 994–1004 (2004).

[28] Anh, D. T., Tanim, A. H., Kushwaha, D. P., Pham, Q. B. & Bui, V. H. Deep learning long short-term memory combined with discrete element method for porosity prediction in gravel-bed rivers. *Int. J. Sedim. Res.* **38**, 128–140 (2023).

[29] Gers, F. A., Schmidhuber, J. & Cummins, F. Learning to forget: continual prediction with LSTM. *Neural Comput.* **12**, 2451–2471 (2000). 11032042 10.1162/089976600300015015

[30] Muhammad, A. U. et al. An autoencoder-based stacked LSTM transfer learning model for EC forecasting. *Earth Sci. Inf.* **16**, 3369–3385 (2023).

[31] Wang, J., Cao, J., Yuan, S., Xu, H. & Zhou, P. Porosity prediction using a deep learning method based on bidirectional spatio-temporal neural network. *J. Appl. Geophys.* **228**, 105465 (2024).

[32] Chadha, G. S., Panambilly, A., Schwung, A. & Ding, S. X. Bidirectional deep recurrent neural networks for process fault classification. *ISA Trans.* **106**, 330–342 (2020). 32684422 10.1016/j.isatra.2020.07.011

[33] Gao, H., Chen, Z. & Li, C. Hierarchical shrinkage multiscale network for hyperspectral image classification with hierarchical feature fusion. *IEEE J. Sel. Top. Appl. Earth Observations Remote Sens.* **14**, 5760–5772 (2021).

[34] Singh, P., Dutta, S. & Pranav, P. Optimizing GANs for cryptography: the role and impact of activation functions in neural layers assessing the cryptographic strength. *Appl. Sci.* **14**, 2379 (2024).

[35] Gao, Z. et al. A Deep-Learning-Based generalized convolutional model for seismic data and its application in seismic Deconvolution. *IEEE Trans. Geosci. Remote Sens.* **60**, 1–17 (2022).

[36] Han, M. E-Bayesian estimations of parameter and its evaluation standard: E-MSE (expected mean square error) under different loss functions. *Commun. Stat. - Simul. Comput.* **50**, 1971–1988 (2021).

[37] Wayo, D. D. K., Irawan, S., Satyanaga, A. & Kim, J. Data-Driven fracture morphology prognosis from high pressured modified proppants based on Stochastic-Adam-RMSprop Optimizers; tf.NNR study. *BDCC* **7**, 57 (2023).

[38] Barakat, A. & Bianchi, P. Convergence and dynamical behavior of the ADAM algorithm for nonconvex stochastic optimization. *SIAM J. Optim.* **31**, 244–274 (2021).

[39] Khan, M. A. et al. A Multi-Attention approach using BERT and stacked bidirectional LSTM for improved dialogue state tracking. *Appl. Sci.* **13**, 1775 (2023).

[40] Sheela, K. G. & Deepa, S. N. Review on methods to fix number of hidden neurons in neural networks. *Math. Probl. Eng.* **2013**, 1–11 (2013).

[41] Zhang, X., Lu, Y. H., Jin, Y., Chen, M. & Zhou, B. An adaptive physics-informed deep learning method for pore pressure prediction using seismic data. *Pet. Sci.* **21**, 885–902 (2024).

[42] Al-Mudhafar, W. J. Incorporation of bootstrapping and cross-validation for efficient multivariate facies and petrophysical modeling. In *SPE Low Perm Symposium 2016* (Society of Petroleum Engineers, 2016).

[43] Wang, G., Ju, Y., Carr, T. R., Li, C. & Cheng, G. Application of artificial intelligence on black shale lithofacies prediction in marcellus shale, appalachian basin. In *SPE/AAPG/SEG Unconventional Resources Technology Conference* (Society of Petroleum Engineers, 2016).

[44] Rahimi, M. & Riahi, M. A. Reservoir facies classification based on random forest and geostatistics methods in an offshore oilfield. *J. Appl. Geophys.* **201**, 104640 (2022).

[45] Pirrone, M., Battigelli, A. & Ruvo, L. Lithofacies classification of thin layered reservoirs through the integration of core data and dielectric dispersion log measurements. In *SPE Annual Technical Conference and Exhibition, ATCE* **3** 2306–2326 (Society of Petroleum Engineers (SPE), 2014).

